# Comprehensive Approaches to Pain Management in Postoperative Spinal Surgery Patients: Advanced Strategies and Future Directions

**DOI:** 10.3390/neurolint17060094

**Published:** 2025-06-18

**Authors:** Dhruba Podder, Olivia Stala, Rahim Hirani, Adam M. Karp, Mill Etienne

**Affiliations:** 1 School of Medicine, New York Medical College, Valhalla, NY 10595, USArhirani2@student.nymc.edu (R.H.);; 2 Department of Neurology, New York Medical College, Valhalla, NY 10595, USA

**Keywords:** postoperative pain, spinal surgery, pain management, multimodal analgesia, regional anesthesia, enhanced recovery after surgery, neuromodulation, personalized medicine, artificial intelligence, opioid-sparing strategies

## Abstract

Effective postoperative pain management remains a major clinical challenge in spinal surgery, with poorly controlled pain affecting up to 50% of patients and contributing to delayed mobilization, prolonged hospitalization, and risk of chronic postsurgical pain. This review synthesizes current and emerging strategies in postoperative spinal pain management, tracing the evolution from opioid-centric paradigms to individualized, multimodal approaches. Multimodal analgesia (MMA) has become the cornerstone of contemporary care, combining pharmacologic agents, such as non-steroidal anti-inflammatory drugs (NSAIDs), acetaminophen, and gabapentinoids, with regional anesthesia techniques, including erector spinae plane blocks and liposomal bupivacaine. Adjunctive nonpharmacologic modalities like early mobilization, cognitive behavioral therapy, and mindfulness-based interventions further optimize recovery and address the biopsychosocial dimensions of pain. For patients with refractory pain, neuromodulation techniques such as spinal cord and peripheral nerve stimulation offer promising results. Advances in artificial intelligence (AI), biomarker discovery, and nanotechnology are poised to enhance personalized pain protocols through predictive modeling and targeted drug delivery. Enhanced recovery after surgery protocols, which integrate many of these strategies, have been shown to reduce opioid use, hospital length of stay, and complication rates. Nevertheless, variability in implementation and the need for individualized protocols remain key challenges. Future directions include AI-guided analytics, regenerative therapies, and expanded research on long-term functional outcomes. This review provides an evidence-based framework for pain control following spinal surgery, emphasizing integration of multimodal and innovative approaches tailored to diverse patient populations.

## 1. Introduction

Postoperative pain management in spinal surgery represents one of the most critical and complex challenges faced by healthcare providers [1,2]. The intricate interplay of surgical trauma, patient-specific factors, and evolving pain pathways necessitates a multidimensional and highly individualized approach [3]. Effective management of postoperative pain is pivotal not only for enhancing patient comfort but also for ensuring optimal recovery outcomes, reducing the risk of chronic pain syndromes, and minimizing reliance on opioids—a crucial consideration amidst an ongoing opioid epidemic [2,4]. Despite substantial advancements in surgical techniques and perioperative care, gaps remain in the implementation of evidence-based pain management protocols that are comprehensive and adaptable to diverse patient needs [1,2,3].

The implications of poorly controlled postoperative pain extend far beyond immediate discomfort. Pain that is inadequately addressed can lead to delayed mobilization, prolonged hospitalization, and increased rates of complications such as venous thromboembolism and pulmonary infections [5,6]. Moreover, unresolved acute pain significantly raises the risk of developing chronic postsurgical pain (CPSP), a debilitating condition that affects 10% to 50% of spinal surgery patients and imposes a considerable burden on patients, caregivers, and healthcare systems alike [3]. Psychological consequences of CPSP, including heightened anxiety and depression, further complicate recovery and hinder the effectiveness of pain management interventions [7,8].

Advancements in spinal surgery, such as minimally invasive surgical techniques and enhanced recovery after surgery (ERAS) protocols, have underscored the need for integrated, multidisciplinary approaches to pain management [2]. The growing recognition of pain as a biopsychosocial phenomenon necessitates strategies that address not only physiological but also psychological and social dimensions of recovery [3,9]. This paradigm shift has fueled interest in multimodal analgesia, which is a cornerstone of contemporary pain management that combines pharmacological and non-pharmacological therapies to target multiple pain pathways. By leveraging the synergistic effects of diverse modalities, multimodal approaches have demonstrated superior efficacy in reducing pain intensity, opioid consumption, and adverse effects [1,2,10,11].

Pharmacological strategies remain central to postoperative pain management, with opioids, non-opioid analgesics, and adjuvants playing key roles. However, the challenges of opioid dependence and tolerance, along with their associated side effects, have spurred efforts to optimize their use through adjunctive therapies and opioid-sparing techniques [3]. Non-opioid analgesics, such as acetaminophen, NSAIDs, and gabapentinoids, have proven effective in addressing specific components of postoperative pain, while adjuvants such as corticosteroids and ketamine offer additional avenues for reducing inflammation and central sensitization [3,5,12].

Non-pharmacological interventions have emerged as integral components of comprehensive pain management plans. Physical therapies, including prehabilitation and early mobilization, aim to enhance functional recovery while mitigating pain through improved circulation and musculoskeletal strength. Psychological interventions, such as cognitive behavioral therapy (CBT) and mindfulness-based stress reduction (MBSR), have shown efficacy in addressing the psychological underpinnings of pain perception and reducing opioid reliance [13,14,15]. Complementary and alternative medicine (CAM) modalities, such as acupuncture, transcutaneous electrical nerve stimulation (TENS), and massage therapy, further expand the repertoire of non-invasive pain management options [1,3].

Innovations in pain management, including regional anesthesia techniques, neuromodulation, and intraoperative analgesic technologies, offer promising avenues for improving outcomes. Regional anesthesia, such as erector spinae plane blocks and epidural analgesia, provides targeted relief while minimizing systemic side effects [6,16]. Neuromodulation therapies, including spinal cord stimulation (SCS) and peripheral nerve stimulation (PNS), have emerged as valuable tools for managing refractory postoperative pain. Intraoperative technologies, such as continuous infusion pumps delivering local anesthetics, represent advancements that directly address pain at its source, offering prolonged relief and enhancing recovery trajectories. Throughout this review, “recovery trajectories” refers to measurable improvements in hospital length of stay, opioid consumption, pain intensity, functional status, and patient satisfaction [1,3,17].

Special populations, including elderly, pediatric, and opioid-tolerant patients, pose unique challenges that necessitate tailored interventions. Elderly patients, with their altered pain thresholds and increased susceptibility to side effects, require careful dose adjustments and close monitoring [7,18]. Pediatric pain management should also prioritize multimodal regimens, combining different classes and routes of analgesics, to maximize pain control while minimizing opioid-related side effects and promoting faster recovery [17]. Opioid-tolerant patients require preoperative optimization and innovative strategies to manage pain effectively while mitigating both the physiological and psychological risks of opioid escalation [19].

The progression of pain management in spinal surgery is continuously shaped by innovation and interdisciplinary collaboration. Emerging therapies such as biologics, regenerative medicine, and nanotechnology-based drug delivery systems show promise for improving postoperative care. For instance, regenerative medicine strategies aim to repair damaged tissues and provide non-pharmacological alternatives for managing chronic and inflammatory pain [20]. Nanotechnology offers targeted delivery of analgesics, potentially reducing side effects and improving efficacy [21]. Technology-driven approaches, including artificial intelligence (AI), machine learning (ML), virtual reality (VR), and augmented reality (AR) are also progressively supplementing pain management paradigms. AI has been shown to improve the accuracy of surgical interventions, minimizing tissue trauma and improving pain outcomes. AI and ML have the potential to enhance perioperative pain management by providing more accurate predictive models and personalized interventions [22]. VR and AR have effective applications in both presurgical planning and supplementing rehabilitation programs. However, significant gaps remain in understanding the long-term efficacy and integration of these novel interventions into clinical practice. Further research is needed to evaluate the reliability, generalizability, effectiveness, and safety of these approaches before their widespread adoption [22].

The management of postoperative pain in spinal surgery demands a comprehensive, patient-centered approach that integrates pharmacological and non-pharmacological strategies. By addressing the physiological, psychological, and social dimensions of recovery, clinicians can enhance outcomes, reduce complications, and improve quality of life for patients undergoing spinal surgery. This review aims to provide a detailed exploration of current practices, emerging innovations, and the unique considerations required for tailoring interventions to diverse patient populations, paving the way for evidence-based and individualized pain management protocols.

## 2. Challenges in Postoperative Pain Management

Postoperative pain following spinal surgery is a significant challenge due to its multifaceted nature. The pain experienced by patients can be severe and is often a combination of incisional pain and deeper tissue pain, including trauma to ligaments, muscles, intervertebral discs, and periosteum [23]. This pain can lead to neural sensitization and the release of inflammatory mediators both peripherally and centrally, complicating pain management [19]. Additionally, patients undergoing spinal surgery frequently have pre-existing chronic pain, which can exacerbate postoperative pain and increase the risk of opioid dependence and tolerance [24]. This population often requires a multimodal analgesic approach to manage pain effectively and reduce opioid consumption.

The complexity of postoperative pain in spinal surgery demands a clinically tailored approach that accounts for each patient’s unique pain profile and comorbidities. Optimizing multimodal analgesic regimens and ensuring sustained long-term outcomes are essential components of delivering effective, patient-centered care and minimizing complications such as chronic pain and opioid dependence [25,26].

### 2.1. Characteristics of Postoperative Pain in Spinal Surgery

Postoperative pain in spinal surgery encompasses nociceptive, neuropathic, and mixed pain components. Nociceptive pain originates from tissue damage incurred during surgery, while neuropathic pain results from nerve injury or compression. These two types of pain often overlap, resulting in a mixed pain presentation that is particularly challenging to treat. The location and severity of the pain depend on various factors, including the surgical approach, the extent and precision of dissection, the use of instrumentation, and patient-specific factors such as pain thresholds and comorbidities [1,7,19,23]. For instance, the anterior approach in spinal surgery, although minimally invasive, often leads to visceral and referred pain, while the posterior approach can result in significant nociceptive pain due to extensive muscle and ligament disruption [12,24]. Additionally, patients undergoing procedures such as spinal fusion or corrective surgeries for deformities report higher pain scores because of the substantial tissue trauma involved. Inflammatory processes that occur during the healing phase amplify nociceptive signals, often leading to hyperalgesia or allodynia [12,24,27]. Patients with pre-existing chronic pain conditions or high opioid tolerance present additional complexities, as their pain is often more difficult to manage and may require tailored multimodal strategies [2].

### 2.2. Risks of Poor Pain Management

The consequences of inadequately managed pain following spinal surgery extend beyond discomfort, significantly impacting recovery, psychological well-being, and healthcare costs. One of the most concerning outcomes of poorly controlled pain is the development of CPSP, which affects up to 50% of spinal surgery patients [3,18]. CPSP is often driven by central sensitization, where prolonged pain signals lead to long-lasting changes in the nervous system, resulting in heightened pain sensitivity even after the initial surgical insult has healed [28].

Poorly controlled acute pain also delays mobilization, a critical component of postoperative recovery. Immobility increases the risk of complications such as deep vein thrombosis (DVT), pulmonary embolism, and respiratory infections, which prolong hospitalization and raise healthcare expenditures. Furthermore, patients with unmanaged pain are more likely to experience reduced satisfaction with their surgical outcomes, contributing to delayed functional recovery and diminished quality of life [8].

Psychological impacts, particularly anxiety and depression, further complicate pain management. These factors can exacerbate pain perception and hinder the effectiveness of therapeutic interventions. For example, patients with heightened anxiety are more likely to experience greater postoperative pain intensities, increasing their risk for developing chronic postsurgical pain and delaying recovery. Inadequate pain control also increases the likelihood of escalated opioid use, leading to tolerance and potential addiction, making long-term opioid dependency another significant risk of poorly managed pain [29].

Neuropathic components of pain, such as referred pain or radicular symptoms from nerve injury or central sensitization, are common after surgery and often do not respond well to standard opioid-based protocols. These pain types typically require specialized interventions, notably anticonvulsants like gabapentin or pregabalin and antidepressants such as duloxetine or venlafaxine, which target neuropathic pathways and have shown benefit in some studies for improving outcomes and quality of life [30,31,32]. Failure to properly manage neuropathic pain can lead to delayed recovery, increased risk of chronic pain, prolonged opioid use, reduced physical function, and higher healthcare costs, significantly increasing the burden on patients and healthcare systems [33].

### 2.3. Addressing the Challenges

Overcoming these challenges necessitates a multifaceted approach. Preoperative assessment to identify high-risk patients, combined with tailored multimodal analgesia strategies, is critical. Multimodal approaches leverage the synergistic effects of pharmacological and non-pharmacological techniques, addressing multiple pain pathways simultaneously [18].

Interdisciplinary collaboration among surgeons, anesthesiologists, and pain specialists ensures comprehensive care. The integration of advancements such as regional anesthesia, neuromodulation, and minimally invasive surgical techniques offers promising solutions, though standardized protocols and further research are needed to fill gaps in current evidence. Personalized, evidence-based pain management strategies have the potential to significantly improve recovery trajectories and overall patient outcomes [34].

In this context, personalized pain management refers to tailoring multimodal analgesia plans to individual risk profiles using validated tools such as the Opioid Risk Tool and Pain Catastrophizing Scale, alongside surgical complexity and opioid history. Implementation strategies include structured preoperative screening, interdisciplinary planning, and use of AI-supported risk prediction models to guide patient-specific analgesic selection and resource allocation [24,26,35,36,37,38,39].

## 3. Pharmacological Approaches to Pain Management

### 3.1. Opioid Analgesia

Opioid medications, including morphine, hydromorphone, fentanyl, oxycodone, and methadone, have historically served as the mainstay for acute postoperative pain management, particularly in procedures associated with severe pain intensity such as spinal surgery [38,40]. They exert their analgesic effects by binding to μ-opioid receptors in the central nervous system, primarily attenuating nociceptive pain signals. While opioids are effective for managing moderate-to-severe pain, their use carries significant risks, including the development of tolerance, physical dependence, opioid-induced hyperalgesia, respiratory depression, sedation, ileus, and other gastrointestinal side effects [41]. There is also a clear association between preoperative opioid use, postoperative dose escalation, prolonged opioid use, and adverse postoperative outcomes, including delayed wound healing, higher readmission rates, and increased mortality [7,42,43]. To mitigate risk, guidelines strongly advocate for limiting the duration and dose of opioid administration by integrating these drugs into multimodal analgesia protocols, which employ agents with varying mechanisms of action to reduce reliance on opioids and their adverse effects [25,44].

These concerns are reflected in formal guidelines from the American Pain Society, ASA, and ASRA, which recommend opioids be reserved for rescue use within a multimodal analgesia framework and discourage routine basal opioid infusions in opioid-naive patients [30].

Patient-controlled analgesia (PCA) is frequently used postoperatively to enable self-titration and optimize opioid dosing, which improves patient satisfaction and helps avoid peaks and troughs in plasma opioid levels compared to intermittent dosing [17]. PCA with morphine, hydromorphone, or fentanyl has demonstrated superior pain control and patient autonomy versus scheduled or as-needed administration, without an increase in side-effect profile [6].

Methadone, a long-acting opioid with NMDA antagonist activity, has been increasingly studied for intraoperative and immediate postoperative use in spinal fusion. Single-dose methadone, administered at induction or early intraoperatively, significantly prolongs analgesia, reduces subsequent intra- and postoperative opioid requirements, and is associated with lower pain scores for up to 72 h postoperatively when compared with shorter-acting opioids or intermittent PCA regimens [45,46,47]. Notably, this opioid-sparing effect may reduce persistent postoperative pain three months after surgery, though ongoing investigation is needed regarding long-term benefits and safety [3].

Epidural and intrathecal opioid administration, by delivering morphine or hydromorphone directly to spinal opioid receptors, can achieve segmental analgesia with lower systemic opioid levels. These techniques have shown efficacy in reducing pain scores for up to 24–48 h, particularly with the use of extended-release formulations such as liposomal or time-release morphine [2,48]. Nevertheless, these modalities carry risks, including delayed respiratory depression, pruritus, and, less commonly, infection or neurologic complications, necessitating careful patient selection and close monitoring [7,48]. Continuous epidural infusion of a local anesthetic and opioid combination can further enhance analgesia and facilitate earlier mobilization, though the risk of motor block, hypotension, and urinary retention must be weighed [7].

### 3.2. Non-Opioid Analgesics

Non-opioid analgesics are foundational in multimodal strategies due to their action on distinct pathways from opioids, thereby providing additive or synergistic analgesic effects and reducing opioid requirements and associated side effects [25,33].

Acetaminophen (paracetamol) is widely used due to its favorable safety profile, antipyretic and mild analgesic effects, and low risk of gastrointestinal or bleeding complications. While acetaminophen alone may be insufficient for moderate to severe postoperative pain after spine surgery, its use in combination with other agents (NSAIDs, opioids) significantly reduces opioid consumption and may improve pain scores [49].

Nonsteroidal anti-inflammatory drugs (NSAIDs), such as ibuprofen, ketorolac, and selective cyclooxygenase-2 inhibitors (COX-2 inhibitors) like celecoxib, effectively reduce both pain and inflammation by inhibiting prostaglandin synthesis [3]. A robust body of evidence demonstrates that perioperative NSAID use confers significant opioid-sparing effects and enhances postoperative pain control after spine surgery [25]. COX-2 inhibitors are often preferred in patients at increased risk of gastrointestinal bleeding, as they preserve gastric protection and platelet function compared to non-selective NSAIDs; however, all NSAIDs should be used judiciously in patients with renal impairment, gastrointestinal ulcers, or cardiovascular disease [1]. Short-term, low-dose NSAID use has not been conclusively shown to interfere with bone healing or increase nonunion risk after spinal fusion, though long-term or high-dose exposure should be avoided in this setting [2].

Gabapentinoids, including gabapentin and pregabalin, are increasingly incorporated into perioperative protocols for their efficacy in reducing neuropathic pain components, decreasing central sensitization, and providing opioid-sparing effects [3,50]. Preoperative administration in particular has been associated with reduced postoperative pain scores and opioid consumption, improved function at three months, and greater patient satisfaction [11,51]. However, these agents can cause sedation, dizziness, or visual disturbances, and must be used cautiously in the elderly or in combination with other sedating drugs [2,3].

Local anesthetics, such as bupivacaine, ropivacaine, and lidocaine, can be employed in several ways: intraoperative infiltration at the surgical site, continuous wound infusion (via catheter), or as part of regional blocks (e.g., thoracolumbar interfascial or erector spinae plane block) [3,17]. Continuous infusion of local anesthetic has been shown to reduce opioid requirements and pain scores in the immediate postoperative period, especially the first 24 h after major spinal procedures [2,17]. Long-acting formulations, including liposomal bupivacaine, offer the potential for extended pain relief beyond the traditional window and can further diminish opioid needs [1,20]. Intravenous lidocaine infusions have also demonstrated benefit for acute pain and possibly for preserving cognitive function after major spine surgery, though findings are mixed [50].

### 3.3. Adjuvant Medications

Adjuvant medications serve as valuable complements to primary analgesics by targeting specific pain pathways or enhancing the effects of other drugs. Corticosteroids, such as intraoperative or perioperative dexamethasone, are sometimes incorporated to reduce surgical inflammation, minimize tissue edema, and decrease postoperative pain and opioid consumption [1,11]. While several studies report beneficial effects—such as decreased pain scores, opioid requirements, and improved range of motion—routine use is limited by concerns regarding hyperglycemia, potential immune suppression, delayed wound healing, and increased risk of infection, especially at higher or repeated dosing [8,11].

Ketamine, a noncompetitive NMDA receptor antagonist, is a valuable adjuvant, especially for opioid-tolerant patients or those with chronic pain syndromes [50]. Perioperative low-dose ketamine infusions have demonstrated significant opioid-sparing effects, reduced pain scores, and attenuation of opioid-induced hyperalgesia after major spine surgery [9,11,52]. Ketamine may be particularly useful in patients at high risk for central sensitization or those with established opioid tolerance, as it acts on pathways implicated in those states [2,52]. The most effective regimens are typically continuous low-dose infusions intraoperatively and sometimes extending into the early postoperative period, though evidence for continuation after surgery is limited by the risk of adverse neuropsychiatric effects [2,53].

Antidepressants, especially serotonin–norepinephrine reuptake inhibitors (SNRIs) like duloxetine, have been shown to modulate pain perception through central descending inhibitory pathways, as well as manage mood disturbances frequently comorbid with chronic pain conditions [33]. Duloxetine, when added to multimodal analgesia regimens, improves acute postoperative pain scores and quality of recovery after major surgery, and may contribute to better long-term pain outcomes [25,33].

Other potential adjuvant medications include α-2 adrenergic agonists (clonidine, dexmedetomidine), magnesium sulfate, journavx, and lidocaine (systemic infusions), though more research is needed to define their specific roles and safety profiles in spinal surgery [25,50].

To contextualize the application of the various multimodal analgesic elements discussed, Table 1 summarizes the major pharmacologic classes used in spinal surgery pain protocols. This includes their mechanism of action, clinical benefits, and potential risks—providing a concise reference for protocol design and personalization across diverse surgical populations.

While pharmacologic agents remain central to multimodal analgesia, non-pharmacologic interventions have become increasingly important in enhancing recovery, reducing opioid reliance, and addressing the biopsychosocial components of postoperative pain. The following section explores these complementary strategies in detail.

## 4. Non-Pharmacological Approaches

### 4.1. Physical Therapy

Physical therapy is a cornerstone of non-pharmacological pain management in postoperative spinal surgery patients. Early mobilization, often initiated within the first 24–48 h post-surgery, improves circulation, reduces pain, and prevents complications such as venous thromboembolism [4,7]. Clinical evidence demonstrates that early physical therapy can decrease hospital length of stay and expedite functional recovery [2,66,67]. In contrast to early postoperative mobilization, prehabilitation focuses on structured, supervised physical conditioning and functional strengthening prior to surgery, with recent studies demonstrating that structured and individualized multimodal programs offer greater efficacy than general or unsupervised exercise.

A scoping review by Gränicher et al. highlighted that prehabilitation programs, which include exercise and education, improve self-reported function, disability, and pain in both the short and long term [68]. Similarly, Eubanks et al. found that prehabilitation programs are feasible and can reduce medical expenditures while improving postoperative pain, disability, self-efficacy, and psychological behaviors [67]. Marchand et al. conducted a randomized clinical trial demonstrating that an exercise-based prehabilitation program for patients awaiting lumbar spinal stenosis surgery resulted in significant improvements in pain intensity, disability, and physical performance measures [69]. Additionally, Lindbäck et al. reported that pre-surgery physiotherapy decreased pain, risk of avoidance behavior, and improved quality of life and physical activity levels before surgery [70].

Programs targeting core and paraspinal muscle strength preoperatively have been shown to improve postoperative outcomes, reducing pain and facilitating faster recovery. Patients engaged in structured prehabilitation programs report fewer postoperative complications and improved mobility [8,68,69,71]. Prehabilitation demonstrated significant quantifiable benefits in lumbar spine surgery, with high-certainty evidence showing reduced preoperative back pain (mean difference −8.20 [95% CI, −8.85 to −7.55]) compared to standard care. Functional outcomes showed moderate-certainty evidence of improvement at 6 months post-surgery (standardized mean difference −2.35 [95% CI, −3.92 to −0.79]), with prehabilitation patients demonstrating superior sit-to-stand performance (12.1 ± 3.6 repetitions vs. 8.4 ± 2.2 in controls). Notably, only 13% of prehabilitation patients reported worsened status compared to 46% in the control group (*p* < 0.01) [8,68,69,71].

The evidence suggests that personalized, supervised prehabilitation may offer the greatest value for patients with high baseline disability or psychosocial risk, supporting its integration into individualized care planning. The integration of physical therapy and prehabilitation into the perioperative care of spinal surgery patients is well supported by evidence indicating improved postoperative outcomes, reduced pain, and enhanced recovery.

The studies cited were selected based on study design (e.g., randomized controlled trials, systematic and scoping reviews), relevance to spine or orthopedic surgery populations, and publication recency. While these studies support the potential benefits of prehabilitation, we also highlight that heterogeneity in outcome measures, patient risk profiles, and program structure may limit generalizability, and some trials report modest or inconclusive findings, especially regarding complication rates and length of stay.

Given its universal application, direct impact on mobility and function, and critical role in preventing postoperative complications such as thromboembolism, physical therapy is widely considered a cornerstone of postoperative care in spinal surgery. While other non-pharmacologic interventions such as cognitive behavioral therapy and mindfulness-based approaches offer important complementary benefits, physical therapy remains the most consistently implemented and foundational non-pharmacologic strategy across patient populations. While early mobilization and prehabilitation are active components of the perioperative plan, inpatient rehabilitation refers to post-discharge care, typically reserved for patients with significant medical or functional needs.

While physical therapy and prehabilitation are essential components of perioperative care, inpatient rehabilitation is not routinely necessary for all patients. Recent evidence suggests that discharge to acute rehabilitation or skilled nursing facilities does not significantly improve outcomes compared to home-based recovery in most spinal surgery populations [72,73]. These findings highlight the importance of individualized patient selection for post-discharge rehabilitation strategies based on preoperative disability, comorbidities, and social support.

### 4.2. Psychological Interventions

Psychological approaches, such as CBT and mindfulness-based interventions, play a significant role in pain management by addressing psychological contributors to pain perception. CBT focuses on altering maladaptive thoughts and behaviors associated with pain, reducing catastrophizing, and enhancing coping strategies. Studies show that CBT effectively decreases pain intensity and reliance on analgesics while improving psychological outcomes. A systematic review found that CBT in the perioperative period may lead to a postoperative reduction in pain and disability in the short-term follow-up compared to care as usual [13].

Mindfulness-based interventions, including guided meditation and relaxation exercises, help patients manage stress and reduce autonomic arousal, which can exacerbate pain. Research highlights reduced opioid consumption and improved psychological outcomes with mindfulness-based interventions. A meta-analysis demonstrated that preoperative mindfulness-based interventions can effectively manage preoperative anxiety and postoperative pain in patients scheduled for elective surgery [14]. Another review indicated that mindfulness-based interventions are feasible and acceptable for use in surgical patient populations, providing preliminary evidence of benefits across a range of surgical contexts [15].

The integration of psychological interventions into perioperative care can enhance pain management and improve overall surgical outcomes. These approaches are particularly beneficial for patients with pre-existing psychological comorbidities or high levels of preoperative anxiety [74].

### 4.3. Complementary and Alternative Medicine (CAM)

CAM techniques, such as acupuncture, transcutaneous electrical nerve stimulation (TENS), and massage therapy, have been increasingly recognized as valuable adjuncts to pain management, particularly in the postoperative setting.

Acupuncture has been shown to stimulate endorphin release and modulate pain pathways, leading to significant reductions in postoperative pain scores [75,76,77,78]. A systematic review and meta-analysis found that patients treated with acupuncture or related techniques experienced less pain and reduced opioid use on the first day after surgery compared to controls [75]. Additionally, a narrative review highlighted the benefits of perioperative acupuncture in reducing postoperative pain and opioid requirements, as well as improving postoperative nausea and vomiting [76].

TENS is a non-invasive modality that disrupts pain signaling through electrical stimulation. The American Pain Society, the American Society of Regional Anesthesia and Pain Medicine, and the American Society of Anesthesiologists recommend considering TENS as an adjunct to other postoperative pain treatments due to its efficacy in reducing pain intensity and opioid consumption without systemic side effects [30]. A systematic review and meta-analysis further supported the effectiveness of TENS in lowering pain intensity and reducing morphine requirements postoperatively [79].

Massage therapy improves circulation, reduces muscle tension, and promotes relaxation, contributing to enhanced recovery and pain relief. Although evidence on the effectiveness of massage therapy is limited, some studies suggest it may reduce pain scores and improve patient satisfaction in the postoperative period [80].

Other CAM practices, such as yoga and tai chi, are emerging as complementary strategies for postoperative rehabilitation. These practices combine physical movement with mindfulness techniques, addressing both physical and psychological aspects of recovery. Preliminary evidence suggests their utility in improving flexibility, reducing pain, and enhancing overall well-being [78]. Taken together, these CAM modalities represent promising, patient-centered strategies that complement pharmacological approaches in postoperative spinal surgery care.

As foundational non-pharmacologic modalities continue to shape recovery protocols, recent advances in regional anesthesia and neuromodulation have further expanded the interventional toolkit for postoperative pain control. These innovative techniques represent a bridge between pharmacologic and procedural approaches and are redefining standards of care in spinal surgery.

## 5. Innovative Pain Management Techniques

### 5.1. Regional Anesthesia

Regional anesthesia techniques have emerged as essential components of postoperative pain management in spinal surgery, offering site-specific analgesia while minimizing systemic opioid exposure and associated side effects. The thoracolumbar interfascial plane (TLIP) block and the erector spinae plane block (ESPB) have emerged as two of the most effective regional anesthesia approaches, demonstrating consistent benefits across recent randomized trials and meta-analyses.

A 2023 network meta-analysis by Hong et al. identified the TLIP block as providing the greatest reduction in postoperative opioid consumption and pain scores when compared to wound infiltration and a control group without block. Specifically, TLIP was associated with a mean opioid reduction of 15 mg (95% CI: −18.8 to −11.2) and significant improvements in pain at multiple postoperative time points. The ESPB also demonstrated strong analgesic efficacy, with no statistically significant difference in opioid reduction when compared to TLIP, highlighting its effectiveness as a comparable alternative [81].

Further supporting the utility of fascial plane blocks, a 2022 scoping review emphasized the growing adoption of ESPB and similar regional techniques in spinal surgery, citing improvements in recovery quality, patient satisfaction, and analgesia [82]. In cervical spine procedures, paraspinal blocks including cervical ESPB and inter-semispinal plane block (ISPB) have been shown to significantly reduce both pain and opioid requirements, expanding the applicability of regional anesthesia beyond lumbar approaches [83].

Recent comparative studies have highlighted potential advantages of ESPB over TLIP in specific contexts. For instance, Peng et al. (2024) and Dilsiz et al. (2024) reported that ESPB resulted in lower opioid consumption and pain scores at 24–48 h postoperatively in lumbar disk surgeries [84,85]. Meta-analyses by Liu et al. and Wu et al. in 2023 and 2024, respectively, confirmed that bilateral ultrasound-guided ESPB significantly decreases perioperative opioid use, prolongs time to rescue analgesia, and reduces opioid-related side effects such as postoperative nausea and vomiting (PONV). These benefits were accompanied by shorter hospital stays and improved patient satisfaction [86,87].

In pediatric populations undergoing procedures like spinal fusion for scoliosis, neuraxial blocks and interfascial plane blocks have demonstrated efficacy in reducing opioid use and enhancing recovery [88]. The use of liposomal bupivacaine (LB), a long-acting formulation, has further advanced regional pain control. Meta-analyses and clinical studies from 2021 to 2025 have shown that LB significantly reduces pain scores and opioid requirements when compared to traditional bupivacaine [89,90,91]. In both adult and adolescent patients, LB has been associated with earlier ambulation, shorter length of stay, fewer emergency visits, and increased reliance on oral rather than intravenous opioids. These outcomes reinforce the role of LB as a critical adjunct within multimodal analgesia protocols.

Ultrasound guidance has enhanced the precision, efficacy, and safety of regional anesthesia techniques. While blocks like ESPB and TLIP are generally considered safe, their effectiveness can be operator-dependent [92]. Overall, the evidence strongly supports the incorporation of ultrasound-guided ESPB and TLIP blocks—particularly those using long-acting local anesthetics like liposomal bupivacaine—into contemporary spinal surgery pain management protocols. Their use not only reduces pain and opioid use but also contributes to earlier mobilization and improved patient-reported outcomes [84,85,86,87].

A comparative summary of the clinical effects of TLIP, ESPB, and related interventions is presented in Table 2 below.

### 5.2. Neuromodulation

Neuromodulation techniques including SCS, PNS, and intrathecal drug delivery systems (IDDS) offer important non-opioid options for managing postoperative and chronic pain following spinal surgery. These therapies modulate pain signaling pathways at the spinal or peripheral level, providing relief for patients with persistent or refractory symptoms.

#### 5.2.1. Spinal Cord Stimulation (SCS)

SCS has emerged as a cornerstone neuromodulation approach for managing chronic postoperative pain, particularly in patients with failed back surgery syndrome (FBSS). A prospective study by Elkholy et al. (2024) showed that SCS reduced median pain scores from 7/10 to 4/10 and significantly decreased opioid use in FBSS patients [93]. These findings align with recommendations from the American Society of Pain and Neuroscience (ASPN), which endorses SCS for chronic refractory low back pain due to its impact on pain reduction and quality of life improvement [94,95].

Although a Cochrane review by Traeger et al. (2023) suggested limited long-term efficacy of SCS in improving pain or function relative to placebo, it acknowledged a modest opioid-sparing effect when used alongside medical therapy [96]. Importantly, the delivery method of SCS may impact outcomes: Goel et al. (2022) found that percutaneous SCS led to shorter hospital stays and fewer readmissions compared to open surgical implantation [97].

Additional evidence supports SCS’s role in improving functional outcomes, including ambulation and mood. Campwala et al. (2021) demonstrated improved physical disability and depression scores in both post-surgical and non-surgical low back pain populations following SCS treatment [98].

#### 5.2.2. Peripheral Nerve Stimulation (PNS)

PNS provides a less invasive alternative to SCS by targeting specific peripheral nerves with subcutaneous electrode placement to modulate nociceptive input. It has been shown to reduce both pain scores and opioid use across several surgical populations, including those undergoing spinal procedures [99,100].

In a multicenter RCT, Ilfeld et al. (2023) demonstrated that PNS resulted in significantly greater reductions in pain and opioid consumption compared to sham controls, with effects lasting several weeks to months postoperatively [101]. Additionally, PNS has been linked to earlier return to function and fewer systemic side effects than opioids or traditional nerve blocks [102]. Common complications—such as lead migration and infection—are rare and typically manageable [103].

The ASPN clinical guidelines support PNS as an effective adjunct in multimodal pain management, particularly for patients with chronic or persistent pain who are poor candidates for SCS [103].

#### 5.2.3. Intrathecal Drug Delivery Systems (IDDS)

IDDS represents another neuromodulatory strategy, delivering analgesics such as intrathecal morphine directly to the spinal cord. Intrathecal opioids provide sustained analgesia for up to 48 h postoperatively, with significant reductions in systemic opioid requirements [54]. Villavicencio et al. (2023) found that combining intrathecal fentanyl and morphine in lumbar fusion patients resulted in superior pain control and lower opioid intake [104].

Recent innovations in IDDS include the addition of adjunctive agents (e.g., bupivacaine, hydromorphone) and programmable pumps that allow for tailored, continuous delivery of multimodal analgesia. These systems have been associated with improved pain control and patient satisfaction while limiting systemic exposure [24]. Additionally, Karavasili et al. (2024) discussed future-forward technologies such as controlled-release platforms for sustained drug delivery, which may enhance precision and prolong analgesic effects in postoperative spinal care [105].

**Figure 1:**
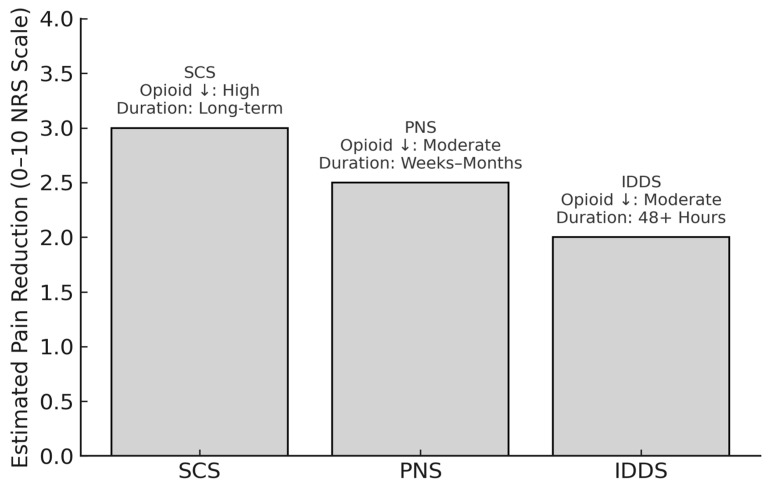
Comparative effectiveness of neuromodulation techniques for postoperative pain management in spinal surgery. ↓: Decreased.

Neuromodulation techniques, including SCS, PNS, and IDDS, are increasingly recognized as integral components of comprehensive postoperative pain management. While SCS offers durable relief and opioid sparing for FBSS and chronic lower back pain, PNS provides a minimally invasive alternative with strong evidence of efficacy. Intrathecal systems, particularly when paired with programmable delivery and multimodal agents, continue to evolve as highly targeted solutions. Collectively, these modalities broaden the therapeutic landscape of spinal surgery, enabling more tailored, durable, and opioid-sparing pain control strategies. To further illustrate their clinical utility and relative distinctions, Figure 1 summarizes the comparative effectiveness of SCS, PNS, and IDDS in terms of pain reduction, opioid-sparing capacity, and analgesic duration. This contextualizes the role of each technique within multimodal pain management frameworks and highlights their respective contributions to personalized perioperative care.

With a broad range of pharmacologic, non-pharmacologic, and interventional options now available, effective postoperative pain management increasingly relies on thoughtful integration. Multimodal analgesia offers a structured framework to combine these elements in a patient-centered, evidence-based manner.

SCS, PNS, and IDDS represent three key neuromodulation strategies for managing postoperative and chronic pain in spinal surgery patients. This figure illustrates estimated average reductions in pain intensity on the 0–10 Numeric Rating Scale (NRS), derived from representative clinical studies, including Elkholy et al. (2024) and Campwala et al. (2021) for SCS [93,98], Ilfeld et al. (2023) and Kaye et al. (2024) for PNS [99,101], and Rawal (2023) and Villavicencio et al. (2023) for IDDS [54,104]. Annotations indicate the relative degree of opioid-sparing, duration of analgesic effect, and device type. These modalities differ in mechanism, risk profile, degree of invasiveness, and ideal patient population, but all contribute meaningfully to opioid-sparing multimodal pain strategies.

Note: Pain reduction estimates are synthesized from recent clinical studies and reviews (2021–2025) and are intended for comparative illustration rather than direct head-to-head inference.

## 6. Multimodal Analgesia

### 6.1. Overview and Clinical Rationale

Multimodal analgesia (MMA) refers to the deliberate and coordinated use of multiple analgesic agents and techniques, each acting on different physiological pathways involved in pain processing. This strategy has become increasingly vital in the context of spinal surgery, where the goal is not only to relieve acute postoperative pain but also to minimize opioid exposure, reduce recovery time, and improve overall patient outcomes. By targeting pain through multiple mechanisms, MMA enhances analgesic efficacy while simultaneously reducing the incidence of opioid-related adverse effects such as sedation, nausea, ileus, and dependence [36].

To enhance the clinical applicability of the multimodal analgesia framework, Table 3 summarizes representative evidence-based regimens from recent randomized controlled trials, network meta-analyses, and large-scale cohort studies. These regimens integrate both pharmacologic and non-pharmacologic components, tailored to surgical context and patient population (e.g., complex spine procedures, pediatric scoliosis fusion, opioid-tolerant or elderly patients). Across multiple studies, triple-agent pharmacologic combinations, typically involving acetaminophen, NSAIDs, and adjuncts such as gabapentinoids or ketamine, have consistently demonstrated superior outcomes over single- or dual-agent strategies. Non-pharmacologic strategies such as early mobilization, patient education, cognitive-behavioral therapy, and regional anesthesia techniques further optimize recovery and reduce opioid reliance. As highlighted in the table, the combination and sequencing of these elements can be adapted to patient risk profiles and procedural complexity, supporting a personalized approach to pain management in spine surgery.

### 6.2. Stepwise Approaches to Pain Management

Pain management strategies in spine surgery can be conceptualized as existing on a spectrum, ranging from single-agent protocols to fully integrated multimodal regimens. At the most basic level, the single-modality approach, typically relying on opioids alone, has long been standard in postoperative care. While opioids are effective for controlling severe nociceptive pain, their use is associated with a well-documented risk of side effects, including respiratory depression, constipation, and the potential for long-term dependency [27,40].

Building on this, dual-agent protocols often combine acetaminophen with NSAIDs to provide additive or synergistic analgesia via prostaglandin inhibition. This combination has demonstrated modest improvements in pain control and a reduction in opioid consumption compared to monotherapy [25]. The next tier in this progression, triple-drug regimens, commonly incorporates a gabapentinoid, such as gabapentin or pregabalin, alongside acetaminophen and NSAIDs. This approach addresses both nociceptive and neuropathic components of surgical pain, offering a more comprehensive pain relief strategy with greater opioid-sparing effects [25].

Standard MMA protocols typically expand further by integrating pharmacologic agents with non-pharmacologic interventions and regional anesthetic techniques. Commonly employed pharmacologic agents include acetaminophen, NSAIDs, and gabapentinoids, often administered alongside regional anesthesia such as the ESPB or TLIP block. Clinical evidence from cohort studies and meta-analyses confirms that these multimodal strategies significantly reduce opioid consumption, decrease postoperative pain scores, shorten hospital stays, and lower complication rates [12,108,110].

Recent innovations have further refined MMA by incorporating regional anesthesia techniques. For instance, the addition of ESPB or TLIP blocks, or the use of liposomal bupivacaine, has been shown to enhance pain control and improve functional recovery. These methods are especially beneficial in minimally invasive procedures such as lumbar fusion, where patients receiving ESPB with liposomal bupivacaine have reported earlier ambulation, shorter hospital stays (2.56 days) and reduced reliance on opioids compared to historical averages for both minimally invasive and open procedures [24,111].

An additional refinement involves the inclusion of continuous infusions of adjunctive agents like ketamine, lidocaine, or dexmedetomidine. These agents provide steady, opioid-sparing analgesia while also minimizing opioid-induced adverse effects. Such protocols are particularly valuable for high-risk patients or those with pre-existing opioid tolerance [37,43].

### 6.3. Evidence-Informed Multimodal Protocols in Spinal Surgery

Structured MMA protocols in spinal surgery have emerged across the perioperative continuum, incorporating pharmacologic and non-pharmacologic elements to optimize analgesia and recovery outcomes. In the preoperative phase, strategies include patient education, expectation setting, and preemptive administration of agents such as acetaminophen, NSAIDs, and gabapentinoids. These interventions serve to reduce central sensitization and enhance postoperative pain control [36,43].

During surgery, intraoperative protocols often incorporate regional techniques like ESPB or TLIP blocks, as well as intrathecal morphine and systemic infusions of ketamine, dexmedetomidine, or lidocaine. These interventions allow for targeted analgesia, reducing the need for systemic opioids and improving hemodynamic stability [12].

Postoperatively, pain management continues with scheduled administration of non-opioid agents, reserving opioids for breakthrough pain. PCA systems may also be used to optimize pain control while empowering patient autonomy [108]. Non-pharmacologic interventions, such as CBT, early mobilization, and structured physical therapy, are increasingly incorporated into multimodal regimens, supporting a holistic, biopsychosocial model of recovery [112].

### 6.4. Challenges and Future Directions

Despite its proven benefits, the adoption of MMA remains inconsistent. Institutional variability, including differences in clinical protocols, provider training, and administrative support, continues to hinder widespread implementation across U.S. healthcare systems [26,39]. Logistical challenges, such as coordinating multi-agent regimens and ensuring interdisciplinary communication, further complicate MMA delivery, especially in high-volume or resource-limited settings [113].

To overcome these barriers, emerging technologies such as AI and ML are being explored for their potential to enhance personalization and streamline decision-making. AI-driven algorithms have demonstrated early promise in predicting postoperative pain trajectories, identifying patients at high risk for prolonged opioid use, and recommending tailored analgesic combinations. However, limitations such as data quality, algorithmic transparency, and ethical considerations must be addressed before these tools can be widely implemented in clinical practice [22,114,115].

Validated tools are increasingly used to identify patients at high risk for poorly controlled postoperative pain or prolonged opioid use. These include the Opioid Risk Tool (ORT), which incorporates psychological, and substance use factors; the Pain Catastrophizing Scale (PCS), which has been shown to predict higher postoperative opioid consumption and pain intensity; and the ASA Physical Status Classification, which correlates with increased perioperative morbidity and long-term opioid use. The Congress of Neurological Surgeons recommends comprehensive preoperative evaluation of opioid use history, such as dose and duration, using standardized definitions, given its strong predictive value for adverse pain outcomes. Additional clinical risk factors such as female sex, tobacco use, depression, anxiety, and poor preoperative function should also be incorporated into risk stratification to guide personalized perioperative planning [116,117,118,119,120].

Parallel research into biomarkers, including molecular, imaging, and neurophysiological indicators, may also pave the way for individualized pain management. These biomarkers could eventually allow clinicians to match specific MMA components to patient-specific pain profiles, leading to more targeted and effective treatment strategies [121].

Multimodal analgesia represents a paradigm shift in perioperative pain management for spinal surgery. By integrating pharmacologic agents, regional anesthesia, and rehabilitative strategies, MMA not only enhances recovery but also mitigates the risks of opioid overuse. Ongoing advancements in AI and biomarker development hold substantial promise for the next evolution of personalized, data-driven pain care.

### 6.5. Integration into ERAS Protocols

ERAS pathway is a multimodal, evidence-based approach designed to optimize perioperative care and improve patient outcomes. In spinal surgery, ERAS protocols incorporate specific analgesic interventions within a broader perioperative care bundle to enhance recovery, reduce opioid consumption, and shorten hospital stays.

Recent recommendations from the ERAS Society, particularly the 2022 lumbar fusion pathway updates, underscore the pivotal role of MMA within the ERAS framework. Core components typically include scheduled administration of acetaminophen, NSAIDs, and gabapentinoids, in conjunction with regional anesthesia techniques such as the ESPB or TLIP block [25,27,40]. These synergistic strategies target nociceptive and neuropathic pain pathways, optimizing analgesic outcomes while mitigating the risks of opioid-related adverse effects.

Clinical studies support the tangible benefits of ERAS protocol integration. A large-scale meta-analysis by Magableh et al. (2024) demonstrated a significant reduction in hospital length of stay (mean difference: –1.41 days) among ERAS-treated spinal surgery patients [122]. Similarly, Ali et al. (2023) reported a sustained decrease in opioid use up to six months postoperatively in patients managed with an ERAS protocol [123]. In terms of functional outcomes, Porche et al. (2022) observed that ERAS patients achieved earlier ambulation, by an average of 0.8 days, compared to those receiving standard perioperative care [124].

The integration of ERAS into spinal surgery not only aligns with contemporary multimodal analgesia strategies but also supports a comprehensive, patient-centered approach to recovery. As institutions increasingly adopt ERAS protocols, continued refinement and adherence to evolving evidence will be essential in standardizing care, improving outcomes, and reducing the burden of postoperative opioid dependence. A summary of current and emerging analgesic protocol types in spinal surgery, including pharmacologic, regional, non-pharmacologic, and AI-driven approaches, is provided below in Table 4.

## 7. Special Populations in Pain Management

### 7.1. Elderly Patients

Postoperative pain management in elderly patients presents unique challenges that require a comprehensive, multimodal approach. Elderly patients present unique challenges in postoperative pain management due to age-related physiological changes, polypharmacy, and comorbidities. Age-associated reductions in renal and hepatic function alter drug metabolism, necessitating careful selection and dosing of analgesics [125,126]. Non-opioid medications, including acetaminophen and COX-2 inhibitors, are preferred for baseline pain management, given their safety profile in older adults [127,128]. Intravenous acetaminophen has demonstrated rapid onset and effectiveness in this population [129].

The aging population experiences increased sensitivity to opioids and is at higher risk for complications such as postoperative delirium, functional decline, and adverse drug effects [130]. To address these challenges, several evidence-based strategies have been identified. A multimodal pain management approach incorporating both pharmacologic and non-pharmacologic interventions is crucial, with emphasis on opioid-sparing techniques [30]. Preoperative assessment of cognitive function and existing chronic pain conditions is essential for developing individualized pain management plans [131]. Regional anesthesia, such as spinal or epidural blocks, is particularly advantageous in elderly patients, reducing systemic opioid exposure and associated risks like respiratory depression. Studies highlight that multimodal analgesia combining regional techniques with acetaminophen can improve recovery and minimize delirium, a common postoperative complication in the elderly [132]. Regional and neuraxial anesthetic techniques have shown particular promise, demonstrating improved pain control while minimizing opioid requirements [133]. When opioids are necessary, careful dose titration starting at 25–50% of standard adult dosing is recommended, with close monitoring for adverse effects [134]. Additionally, certain medications should be avoided or used with extreme caution in elderly patients, including long-acting opioids, meperidine, and tramadol, due to their increased risk of adverse effects in this population [135].

### 7.2. Pediatric Patients

Pediatric patients undergoing spinal surgery, such as posterior spinal fusion for scoliosis, experience severe acute postoperative pain, requiring tailored approaches [136]. Multimodal analgesia is the standard, integrating neuraxial blocks, low-dose opioids, and non-opioid adjuvants like gabapentin [137]. Regional anesthesia, including caudal and epidural techniques, effectively reduces opioid consumption and enhances recovery [138]. Low-dose ketamine has also emerged as a valuable adjunct in opioid-tolerant or high-risk pediatric patients, as evidence shows that ketamine reduces opioid-induced hyperalgesia and improves postoperative pain control [139,140,141]. Careful monitoring is crucial to avoid potential side effects, ensuring optimal outcomes in this vulnerable population.

Additionally, the implementation of rapid recovery pathways incorporating early mobilization and multimodal analgesia has been shown to lead to decreased hospital stays and improved pain control [142]. The evidence suggests that a comprehensive approach combining appropriate opioid use with multiple non-opioid analgesics, regional anesthesia techniques, and standardized recovery protocols optimizes pain control while minimizing complications in this patient population.

### 7.3. Opioid-Tolerant Patients

Managing post-operative pain in opioid-tolerant patients requires a nuanced and comprehensive approach. These patients typically experience higher pain scores and require significantly larger opioid doses compared to opioid-naive individuals, requiring strategies that address increased opioid requirements while mitigating withdrawal risks [143].

One of the primary challenges is that opioid-tolerant patients typically require higher doses of opioids for acute pain management while paradoxically being at increased risk for opioid-induced ventilatory impairment. As Macintyre et al. note, these patients develop differential tolerance to opioid effects, with tolerance to analgesia developing faster than tolerance to respiratory depression [52]. This creates a concerning clinical scenario where patients need more opioids for pain control but face greater risks from those higher doses.

Another significant challenge is the increased risk of postoperative complications. Research has shown that preoperative opioid use is associated with higher rates of infection, longer hospital stays, increased healthcare costs, and higher readmission rates [144]. Additionally, these patients often report higher pain scores and use greater amounts of opioids postoperatively compared to opioid-naïve patients [145].

Several evidence-based strategies have emerged for managing these challenges. A multimodal analgesic approach is strongly recommended, incorporating non-opioid analgesics, adjuvant medications, and regional anesthetic techniques. The use of ketamine as an adjuvant has shown particular promise, with studies demonstrating its ability to reduce postoperative opioid requirements in opioid-tolerant patients [146]. Multimodal analgesia is particularly effective, incorporating ketamine, lidocaine, and α-2 agonists like clonidine to provide non-opioid pain relief. Low-dose ketamine infusions have demonstrated significant reductions in opioid consumption and improved pain control without increasing adverse effects [139].

Preoperative planning is essential, including assessing baseline opioid use and establishing patient-specific tapering plans. ERAS protocols tailored for opioid-tolerant patients have shown promise in improving outcomes while reducing postoperative opioid dependence [147]. Intrathecal and epidural analgesia also play a critical role, offering targeted pain relief with fewer systemic side effects [54]. Care coordination through specialized services like transitional pain services or perioperative surgical homes has also proven beneficial. These programs provide comprehensive management from preoperative planning through post-discharge care, helping to optimize outcomes and reduce complications [148].

Successful management of postoperative pain in opioid-tolerant patients requires careful balancing of analgesic efficacy and safety concerns, along with coordinated care approaches and multimodal pain management strategies. Future research should focus on developing more targeted approaches for this high-risk population.

## 8. Future Directions in Pain Management

### 8.1. Emerging Therapies

Biologics, including platelet-rich plasma (PRP), platelet-rich fibrin (PRF), and mesenchymal stem cells (MSCs), represent a novel approach to pain management, particularly in postoperative spine surgery patients [149]. These therapies aim to promote tissue repair and regeneration, offering a shift from traditional pain management strategies that focus solely on symptom relief. PRP and PRF, derived from a patient’s blood, contain growth factors that can stimulate healing and reduce inflammation, potentially addressing the root cause of pain. Studies such as Bhatia and Chopra (2016) have demonstrated significant reductions in pain scores following PRP injections for chronic disc prolapse, supporting their short-term efficacy [150].

The use of MSCs, sourced from bone marrow or adipose tissue, has also shown promise. These cells have the ability to differentiate into various types of cells, which could aid in regenerating damaged intervertebral discs. A study by Amirdelfan et al. (2021) using allogeneic MSCs reported significant pain relief compared to controls, though the long-term effects on disc structure remain unclear [151]. Additionally, bioengineered devices for annulus fibrosus repair, such as Barricade and Anulex-Xclose, aim to prevent recurrent herniation and support disc stability. While these devices have shown success in reducing reherniation rates, further research is needed to understand their role in promoting true tissue regeneration [149].

Nanotechnology represents another frontier in pain management, offering targeted drug delivery systems that minimize systemic exposure and side effects. Nanoparticles engineered to carry anti-inflammatory drugs or analgesics can localize treatment to specific sites, thereby enhancing efficacy. In preclinical trials, nanotechnology-based delivery systems have shown promise in reducing chronic inflammation and managing postoperative pain with fewer complications [152].

Recent experimental studies have validated the therapeutic potential of nanotechnology in pain management. Notably, extended-release liposomal formulations such as Exparel™ and Zynrelef™ have demonstrated significant efficacy in clinical contexts. Exparel™ is a bupivacaine-based anesthetic encapsulated in multivesicular liposomes designed to slowly release the drug over 72 h at the surgical site, providing prolonged postoperative analgesia. Zynrelef™, a combination of bupivacaine and meloxicam, utilizes a similar delivery system to provide both local anesthesia and anti-inflammatory effects for up to 72 h, approved for use in various soft tissue and orthopedic surgeries [153]. Additionally, nanotechnology-based patches such as Kailo™ and NeuroCuple™ represent a non-pharmacologic approach. These patches contain nanocapacitors—electrically conductive particles theorized to interact with the body’s natural electrical signals—to modulate nerve signaling and reduce pain perception. Early clinical trials have shown that their use can reduce pain intensity and opioid consumption following joint replacement surgeries and in chronic pain patients [154]. Complementary preclinical research supports the use of engineered nanoparticles to encapsulate and deliver analgesics or anti-inflammatory agents directly to affected tissues, enhancing site-specific efficacy while reducing systemic side effects [155]. Together, these experimental and clinical advances position nanotechnology as a highly promising frontier in targeted, multimodal pain therapy.

### 8.2. Technology-Driven Innovations

The landscape of postoperative pain management in spine surgery has been fundamentally transformed by a wave of technological innovations. These advancements, spanning AI, ML, AR/VR, advanced navigation systems, and novel biomaterials, have collectively enhanced the precision, safety, and personalization of perioperative and postoperative care. This subsection reviews the most impactful technological developments, their clinical implications, and the evidence supporting their integration into spine surgery practice.

AI and ML have emerged as pivotal tools in optimizing postoperative pain management by enabling predictive analytics, risk stratification, and individualized care pathways. ML algorithms can process vast datasets to identify patients at higher risk for postoperative complications, including severe pain, opioid dependence, and delayed recovery. For example, ML models have demonstrated high accuracy in predicting surgical candidacy and postoperative complications, outperforming traditional risk assessment tools such as the American Society of Anesthesiologists (ASA) physical status classification [156,157]. In a large multicenter study, ML-based models achieved up to 92.1% accuracy in predicting lumbar spine surgical candidacy and were able to forecast postoperative complications with greater specificity and sensitivity than conventional methods [158]. These predictive capabilities allow clinicians to tailor analgesic regimens, anticipate the need for multimodal pain strategies, and allocate resources for high-risk patients, thereby reducing the incidence and severity of postoperative pain.

Recent experimental findings further support the clinical utility of AI in postoperative pain prediction and management. A 2024 pilot study demonstrated that a machine learning model analyzing facial expressions outperformed traditional physiological markers—such as vital signs and the analgesia nociception index—in predicting severe postoperative pain, achieving an AUROC of 0.93 for identifying patients with high pain scores (NRS ≥ 7) after gastrectomy [159]. Additionally, a narrative review incorporating experimental validation emphasized that ML-driven models can accurately identify modifiable risk factors for persistent opioid use and chronic postoperative pain, advocating for their integration into targeted perioperative pain strategies [22].

Furthermore, AI-driven image analysis and intraoperative guidance have improved the accuracy of surgical interventions, indirectly contributing to better pain outcomes. Automated segmentation of intraoperative imaging and identification of anatomical landmarks using ML have achieved up to 99% accuracy, reducing the likelihood of surgical errors that can lead to increased postoperative pain [160]. By minimizing intraoperative trauma and optimizing implant placement, these technologies help decrease tissue injury and subsequent pain, supporting enhanced recovery protocols.

AR and VR technologies have revolutionized both intraoperative and postoperative phases of spine surgery. Intraoperatively, AR provides real-time visualization of anatomical structures and surgical plans, improving the accuracy of pedicle screw placement and reducing the risk of nerve injury—a common source of postoperative pain [161,162]. Studies have shown that AR-assisted procedures result in higher screw placement accuracy and lower cortical breach rates compared to freehand techniques, with associated reductions in operative time and radiation exposure [162,163]. These improvements translate into fewer postoperative complications and less pain for patients.

VR, while primarily used in preoperative planning and surgical training, is increasingly being explored as an adjunct for rehabilitation programs. These VR-based programs facilitate early mobilization and patient engagement, both of which are critical for minimizing chronic pain development after spine surgery [164]. The integration of AR and VR with navigation systems, such as the 7D Surgical platform, further enhances intraoperative precision and postoperative outcomes by providing continuous, patient-specific feedback [165].

Neuromodulation continues to evolve, with high-frequency SCS and dorsal root ganglion stimulation emerging as promising options for managing chronic and postoperative pain. Wireless devices utilizing nanotechnology have minimized complications associated with traditional SCS systems, such as bulky implantable pulse generators. These innovations allow for minimally invasive procedures with enhanced patient compliance and reduced healthcare costs [166].

### 8.3. Research Gaps and Opportunities

Despite these advancements, significant research gaps remain in understanding the long-term efficacy and safety of emerging therapies. Biologics and nanotechnology require robust clinical trials to validate their effectiveness and address potential risks. Accessibility and cost-effectiveness of cutting-edge neuromodulation technologies, particularly in resource-constrained settings, present additional challenges [167,168,169].

Integrating multimodal analgesia with technology-driven innovations represents a promising area for future research. Collaborative efforts among clinicians, engineers, and policymakers are essential to develop standardized protocols and address regulatory hurdles. Moreover, the role of AI in tailoring pain management strategies warrants further exploration, especially in predicting patient-specific risks and benefits [114,115,170].

## 9. Conclusions

Postoperative pain management in spinal surgery is undergoing a necessary transformation. Historically dominated by opioid-centric models, the field is now largely defined by multimodal strategies that are safer, more effective, and increasingly patient-centered. Multimodal analgesia, integrating pharmacologic, regional, and non-pharmacologic interventions, has consistently demonstrated superiority in reducing pain intensity, minimizing opioid consumption, and accelerating functional recovery. Its growing adoption marks a paradigm shift from reactive to proactive perioperative care.

Key innovations, including liposomal bupivacaine, ultrasound-guided regional blocks, and continuous infusions of agents like ketamine and dexmedetomidine, have expanded the therapeutic toolkit. Neuromodulation techniques such as SCS and PNS are also emerging as powerful adjuncts, particularly in cases of chronic or refractory postoperative pain. The integration of these approaches into ERAS pathways has yielded measurable improvements in opioid stewardship, hospital length of stay, and time to ambulation, reinforcing the clinical value of coordinated, protocol-driven care.

Looking ahead, the personalization of pain management represents the next frontier. AI-driven prediction models and biomarker-guided interventions are poised to optimize treatment selection and improve outcomes across diverse patient populations. However, some challenges remain, including institutional variability, implementation barriers, and disparities in access to advanced therapies, particularly in resource-constrained areas.

The biopsychosocial dimensions of recovery require equal attention, with evidence supporting the integration of cognitive behavioral approaches and mindfulness-based interventions. Standardizing best practices while maintaining flexibility for patient-specific customization is crucial, as demonstrated by successful multidisciplinary transitional pain services. Special populations, including elderly and opioid-tolerant patients, require carefully tailored approaches, with emerging evidence supporting modified protocols that account for altered pharmacokinetics and increased vulnerability to complication.

This review provides a comprehensive framework for evidence-based, multimodal pain management in spinal surgery. By uniting traditional pharmacologic strategies with emerging technologies and patient-specific customization, clinicians can significantly improve recovery trajectories and reduce the burden of opioid-related complications. The future of spine surgery pain management lies in the seamless integration of AI-guided analytics, regenerative therapies, and personalized intervention protocols. As the field continues to evolve, ongoing research, interdisciplinary collaboration, and systems-level integration will be critical to achieving durable, equitable, and individualized pain care.

## Figures and Tables

**Table 1:** Summary of pharmacological agents in multimodal analgesia for spinal surgery.

Class/Agent	Role and Mechanism	Key Evidence/Effects	Limitations/Risks
Opioids (morphine, hydromorphone, oxycodone, methadone) [45]	Mainstay for moderate-to-severe nociceptive pain; μ-opioid receptor agonists	High efficacy in acute pain. Methadone has prolonged analgesia and may reduce opioid needs post-discharge. PCA preferred.	Respiratory depression, OIH, tolerance, ileus, dependence, tolerance
Epidural/Intrathecal Opioids [54]	Central/segmental pain blockade	Effective for multilevel surgery. Morphine provides longer relief; fentanyl acts faster. Suitable for continuous infusion or single shot.	Respiratory depression, pruritus, urinary retention, rare neuraxial complications. Best for high-pain-risk patients.
NSAIDs/COX-2 Inhibitors [55]	Prostaglandin synthesis inhibitors (COX inhibition); analgesic and anti-inflammatory	Meta-analyses support opioid-sparing effects and better pain control. COX-2 preferred in high-bleeding-risk patients.	GI bleeding, renal impairment, bleeding risk (less with COX-2), possible impaired fusion at high doses
Acetaminophen (IV or oral) [30,56]	Central prostaglandin inhibition	Effective multimodal adjunct. IV offers faster onset; oral and IV equally effective after repeat dosing.	Hepatotoxicity with overdose. Mild opioid-sparing effect when used alone.
Gabapentinoids (gabapentin, pregabalin) [30,57]	α2δ calcium channel modulators; reduce central sensitization	Decrease opioid use and pain scores; useful perioperatively. Some data on long-term benefit.	Sedation, dizziness, risk of respiratory depression (esp. with opioids), misuse risk; dose-adjust for renal impairment
Local Anesthetics (infiltration, catheter, block, IV lidocaine) [1,2,3,17]	Sodium channel blockers; inhibit nociceptive transmission	Effective in ESPB, wound catheters, and IV use. Reduce pain and opioid use. IV lidocaine may aid cognition.	Systemic toxicity if overdosed, local site failure, rare cardiac/CNS events. Monitoring needed.
Ketamine [2,50,52,53]	NMDA receptor antagonist; blocks central sensitization	Reduces opioid need, effective in opioid-tolerant or chronic pain patients. Low-dose infusions preferred.	Hallucinations, dysphoria, nausea, hypertension. Abuse potential at high doses.
α2 Agonists (dexmedetomidine, clonidine) [25,50]	Central α2 activation; sedative and analgesic adjuvant	Enhance sedation and analgesia; reduce opioid needs. Useful in neuraxial and systemic regimens.	Bradycardia, hypotension, sedation
Magnesium Sulfate [58,59,60]	NMDA antagonist	Emerging data show opioid-sparing when used perioperatively	Hypotension, flushing, respiratory depression at high doses
Antidepressants (SNRIs, TCAs, e.g., duloxetine) [25,33]	Central modulation of pain; treat neuropathic pain	Reduce opioid use and improve mood. Mostly used as adjuncts in perioperative care.	Sedation, nausea, interactions; slow onset of action
Corticosteroids [61,62]	Anti-inflammatory, reduce local edema	Some support for use in high-inflammatory spine procedures; short-term use only	Hyperglycemia, infection risk, wound issues, psychiatric effects
Other Adjuncts (melatonin [35], vitamin C [63], cannabinoids [64,65])	Various proposed analgesic pathways	Preliminary data for pain and opioid reduction, mostly experimental	Sparse evidence, unknown safety in spine surgery

Legend: PCA: Patient-Controlled Analgesia; OIH: Opioid-Induced Hyperalgesia; COX: Cyclooxygenase; IV: Intravenous; SNRIs: Serotonin–Norepinephrine Reuptake Inhibitors; TCA: Tricyclic Antidepressants; ESPB: Erector Spinae Plane Block.

**Table 2:** Comparative effectiveness of regional anesthesia techniques in spinal surgery.

Block Type/Intervention	Opioid Reduction	Pain Score Reduction	Other Benefits	Notes
TLIP [81]	↓ 15 mg (MD −18.8 to −11.2)	Significant at all postoperative time points	Effective for lumbar procedures	2023 network meta-analysis
ESPB (Lumbar) [86,87]	Similar to TLIP	Effective at 24–48 h	↓ PONV, ↑ satisfaction, ↓ length of stay	Preferred for ease and safety
ESPB (Cervical) [83]	MD: −1.37 mg	Effective (de Liyis et al.)	Extends utility to cervical spine	Includes inter-semispinal plane block (ISPB)
LB + ESPB/TLIP [91]	MD: −0.42 mg	MD: −0.31	↑ ambulation, ↓ ED visits, ↓ IV opioid use	Enhanced in multimodal protocols
Ultrasound Guidance [92]	N/A	N/A	↑ block accuracy, ↓ complications	Operator dependent but considered standard care

Legend: TLIP: Thoracolumbar Interfascial Plane Block; ESPB: Erector Spinae Plane Block; LB: Liposomal Bupivacaine; PONV: Postoperative Nausea and Vomiting; ED: Emergency Department; IV: Intravenous; MD: Mean Difference. ↑: Increased. ↓: decreased. All findings based on data from randomized trials and systematic reviews published between 2021 and 2025.

**Table 3:** Common evidence-based multimodal analgesia regimens tailored to specific spinal surgery populations. Clinical benefits include opioid-sparing effects, improved pain scores, earlier ambulation, and reduced length of stay. Based on high-level evidence including RCTs, meta-analyses, and multi-institutional cohort studies (2020–2025).

Patient Population/Context	Pharmacologic Components	Non-Pharmacologic Components	Clinical Benefits
Adult Spine Surgery—General [25,106]	Acetaminophen + NSAID + Gabapentinoid (±Ketamine)	Early mobilization	↓ Morphine use by 26 mg, ↓ pain score by 2.3/10 at 24 h
Adult Lumbar Fusion [107]	Ketorolac + Orphenadrine + Gabapentin	ERAS protocol; early PT	↓ Opioid use and pain scores without ↑ LOS
Adult Lumbar Fusion [12,37]	Ketamine + Dexmedetomidine + ESPB or Intrathecal Morphine	Early mobilization + CBT	↑ Pain control, ↓ opioid need, better tolerated in high-risk patients
Elderly/Opioid-Tolerant [24]	Gabapentinoids (reduced dose) + NSAIDs + Regional anesthesia (e.g., ESPB)	Prehabilitation; gradual mobilization	↓ Opioid escalation ↓ delirium risk Individualized dosing
Pediatric/Adolescent Scoliosis Fusion [108,109]	Acetaminophen + NSAID + Gabapentinoid + Methadone or Remifentanil ± Dexmedetomidine	Regional anesthesia (liposomal bupivacaine) + early ambulation	↓ Opioid use, ↑ ambulation ↓ LOS, Pain scores maintained

↑: Increased; ↓: Decreased.

**Table 4:** Classification of analgesia protocols in spinal surgery.

Protocol Type	Brief Description
Single-Modality [27,40]	Use of a single agent, typically opioids. Effective but high risk for adverse effects.
Double-Drug [25]	Combines two agents (e.g., acetaminophen + NSAIDs) for synergistic pain control.
Triple-Drug [25]	Adds gabapentinoids to double-drug regimens for improved neuropathic pain coverage.
MMA [12,27,72,108,109,110]	Integrates ≥ 3 agents from different classes (e.g., acetaminophen, NSAIDs, gabapentinoids) ± regional blocks for opioid-sparing pain control.
MMA + Regional Blocks [12,24,37,43,111]	MMA enhanced with ESPB, TLIP, or liposomal bupivacaine for targeted relief and faster recovery.
MMA + Continuous Infusions [37,43]	Adds ketamine, lidocaine, or dexmedetomidine infusions to MMA for opioid-tolerant or high-risk patients.
MMA + Non-Pharmacologic Adjunct [112]	Incorporates CBT, early mobilization, or physical therapy for functional and psychosocial optimization.
Intrathecal Analgesia [12,25]	Delivers opioids or adjuncts directly to the spinal cord; useful in major or prolonged surgeries.
Preemptive Analgesia [36,43]	Administered preoperatively to reduce central sensitization and postoperative pain.
AI-Driven Personalized MMA [22,114,115]	Uses AI algorithms or biomarkers to predict pain responses and tailor analgesic regimens; an emerging frontier.

Legend: MMA: Multimodal Analgesia; ESPB: Erector Spinae Plane Block; TLIP: Thoracolumbar Interfascial Plane Block; AI: Artificial Intelligence; CBT: Cognitive Behavioral Therapy. Protocol classifications are based on randomized controlled trials, systematic reviews, and expert recommendations from 2020–2025.
